# Mental Workload Alters Heart Rate Variability, Lowering Non-linear Dynamics

**DOI:** 10.3389/fphys.2019.00565

**Published:** 2019-05-14

**Authors:** Stéphane Delliaux, Alexis Delaforge, Jean-Claude Deharo, Guillaume Chaumet

**Affiliations:** ^1^Aix Marseille Univ, INSERM, INRA, C2VN, Marseille, France; ^2^Pôle Cardio-Vasculaire et Thoracique, Service des Explorations Fonctionnelles Respiratoires, AP-HM, Hôpital Nord, Marseille, France; ^3^Service de Médecine et Santé au Travail, AP-HM, Hôpital de la Timone, Marseille, France; ^4^Pôle Cardio-Vasculaire et Thoracique, Service de Cardiologie, AP-HM, Hôpital de la Timone, Marseille, France; ^5^AltraBio, Lyon, France

**Keywords:** mental workload, heart rate variability, non-linear dynamics, correlation dimension, cardiovascular function, arrhythmia

## Abstract

Mental workload is known to alter cardiovascular function leading to increased cardiovascular risk. Nevertheless, there is no clear autonomic nervous system unbalance to be quantified during mental stress. We aimed to characterize the mental workload impact on the cardiovascular function with a focus on heart rate variability (HRV) non-linear indexes. A 1-h computerized switching task (letter recognition) was performed by 24 subjects while monitoring their performance (accuracy, response time), electrocardiogram and blood pressure waveform (finger volume clamp method). The HRV was evaluated from the beat-to-beat RR intervals (RRI) in time-, frequency-, and informational- domains, before (Control) and during the task. The task induced a significant mental workload (visual analog scale of fatigue from 27 ± 26 to 50 ± 31 mm, *p* < 0.001, and NASA-TLX score of 56 ± 17). The heart rate, blood pressure and baroreflex function were unchanged, whereas most of the HRV parameters markedly decreased. The maximum decrease occurred during the first 15 min of the task (P1), before starting to return to the baseline values reached at the end of the task (P4). The RRI dimension correlation (D2) decrease was the most significant (P1 vs. Control: 1.42 ± 0.85 vs. 2.21 ± 0.8, *p* < 0.001) and only D2 lasted until the task ended (P4 vs. Control: 1.96 ± 0.9 vs. 2.21 ± 0.9, *p* < 0.05). D2 was identified as the most robust cardiovascular variable impacted by the mental workload as determined by posterior predictive simulations (*p* = 0.9). The Spearman correlation matrix highlighted that D2 could be a marker of the generated frustration (*R* = –0.61, *p* < 0.01) induced by a mental task, as well as the myocardial oxygen consumption changes assessed by the double product (*R* = –0.53, *p* < 0.05). In conclusion, we showed that mental workload sharply lowered the non-linear RRI dynamics, particularly the RRI correlation dimension.

## Introduction

Studies of work and its mental consequences raised the concept of mental workload that has been initially defined by Gopher and Donchin (1986): “Mental workload may be viewed as the difference between the capacities of the information processing system that are required for task performance to satisfy performance expectations and the capacity available at any given time.” According to Hart and Staveland (1988), the developer of the NASA Task Load Index (NASA-TLX), workload can be defined as “the cost incurred by a human operator to achieve a particular level of performance” (p. 2) that can have deleterious consequences on health.

The impact of mental workload on health has been widely investigated, particularly the links between mental workload and cardiovascular diseases. Job-strain (high job demands and/or low decision latitude) is found to raise the risk of coronary heart disease (Bosma et al., 1997; Kivimäki et al., 2002, 2012; Netterstrom et al., 2006), as well as hypertension (Markovitz, 1993; Schwartz et al., 1996). However, no absolute causal demonstration has emerged from the studies to date. High mental workload raises heart rate and blood pressure (Schnall et al., 1990, 1994; Wilson, 1992, 1993; Veltman and Gaillard, 1996; Hankins and Wilson, 1998; Wilson and O’Donnell, 1988; Hjortskov et al., 2004), two indexes of cardiovascular risk. Subtler cardiovascular modifications, also linked to an increased cardiovascular risk, have been described as a result of heart rate variability analysis (HRV). HRV is a highly sensitive marker of clinical status, especially of cardiac disease and autonomic neuropathy (Task Force of the European Society of Cardiology and the North American Society of Pacing and Electrophysiology, 1996). Furthermore, HRV has been successfully used to assess environmental or physiological conditions likely to modify sympathetic-parasympathetic balance even without any cardiac disease or autonomic neuropathy. HRV is lowered by mental effort and mental workload (Mulder and Mulder, 1981; Hjortskov et al., 2004; Wang et al., 2005) probably due to sympathetic activation and/or to parasympathetic withdrawal (Hatch et al., 1986; Grossman and Svebak, 1987; Grossman, 1992; Hjortskov et al., 2004; Collins et al., 2005; Wang et al., 2005; Taelman et al., 2011). According to more recent studies, mean RR interval (RRI) appears to be a most sensitive measure (Capa et al., 2008a,b; De Rivecourt et al., 2008; Henelius et al., 2009; Weippert et al., 2009) even if there is a strong inverse relationship (Hennessy et al., 2001; Sandercock et al., 2005; Fu et al., 2006; Paul-Labrador et al., 2006) between mental effort and HRV power. Few studies have searched for links between mental workload and baroreflex function while arterial baroreflex is a major determinant of the sympatho-vagal balance and of the neurally mediated HRV supported by the autonomic nervous system (ANS). Mental workload seems to reduce the baroreflex sensitivity (Mulder and Mulder, 1981) as exhibited in most cardiovascular diseases and identified as an independent and robust cardiovascular risk factor (Rovere et al., 1998).

But HRV analysis trough time- and frequency- domains mainly focus on sympatho-vagal balance assessment (even for baroreflex assessment) which does not completely and robustly describe the complex time behavior of heart rhythm. The lack of a clear ANS unbalance during mental stress, and the need of exploring different metrics (e.g., non-linear or multivariate) than standard time and frequency domain measures, have been reported recently (Vuksanović and Gal, 2007; Melillo et al., 2011; Widjaja et al., 2015; Faes et al., 2016). Non-linear analysis technics and metrics try to catch non-linear dynamics properties that characterize complex systems. Heart rate fluctuations (RRI time series) are the result of a complex process including a complex system that is the heart (Goldberger, 1996, 2002) inside the human body and several influencing environmental factors. These recent tools seem to be more sensitive and precise to describe cardiac and clinical status and to assess the associated prognosis in different diseases (Huikuri et al., 2008). Recent studies have demonstrated relationships between mental workload and mental fatigue and HRV. The first study (Mukherjee et al., 2011) highlighted the sensitivity and reliability of HRV time domain (mean RRI), frequency domain (high and low frequency powers), and non-linear indexes (Poincaré estimates and detrended fluctuation analysis indexes) to mental effort tasks in healthy seniors. The second study (Gergelyfi et al., 2015) investigated HRV, as well as electroencephalogram (EEG), eye blink and skin conductance variables during a mental fatigue induced task (Sudoku) in a protocol dedicated to discriminate between motivational state and mental fatigue. Both studies showed the interest, the sensitivity and the reliability of the non-linear Poincaré plot analysis. Recent non-linear metrics have been developed to characterize fractal time behavior, autosimilarity, or complexity of the time series. Among them, RRI dimension correlation (D2) is considered to be a marker of the cardiovascular system adaptability assessing its degree of freedom. As Tako-Tsubo syndrome is a brutal heart failure that is associated with rhythmic troubles and often follows a severe emotional stress (Sharkey et al., 2010), including extreme cases of overworking (Akashi et al., 2008), and as D2 has been shown to be the more precocious and sensitive cardiovascular parameter in the prediction of ventricular fibrillation occurrence in high-risk subjects (Skinner et al., 1993), we tested the hypothesis that D2 could be a monitoring index of the cardiovascular impact of mental workload intensity induced by a switching task.

In this study, we aimed to investigate cardiovascular function changes during a fatiguing mental task, focusing on HRV and particularly its non-linear properties, but also on spontaneous arterial baroreflex, a robust independent cardiovascular risk factor modulating sympatho-vagal balance, and on myocardial oxygen consumption that reflect cardiac metabolic constraint and cardiac stress. The main objective was to identify relevant cardiovascular indexes of mental workload and document their respective time course. We specifically targeted the correlation dimension (D2) of the RRI.

## Materials and Methods

### Subjects

**FIGURE 1:**
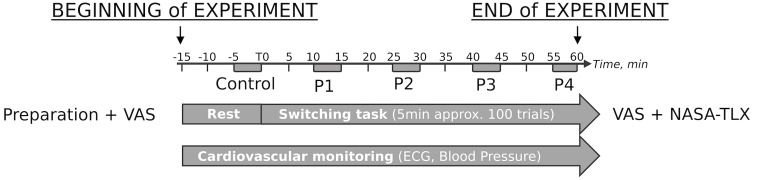
Protocol design. The protocol lasted approximately 1 h 20 min, including 15 min under resting conditions (calm, room temperature control, sitting position in front of a computer, no visual stimulus), of which the last 5 min were used as the Control conditions, and 60 min of the switching task on the computer. Before the task started the visual analog scale (VAS) was administered and VAS + NASA-TLX questionnaires were completed immediately after the task ended. During the task, the last 5 min of each temporal quartile (i.e., 25, 50, 75, and 100% of task duration, respectively) were used for cardiovascular function assessment and denoted by P1, P2, P3, and P4, respectively.

Power analysis was performed to determine sample size. Sixteen number of pairs are necessary to achieve a power of 80% and a level of significance of 5% (two sided), for detecting an effect size of 0.8 between pairs. Accordingly, twenty-four healthy volunteers participated in the study. The subjects were orally checked by a medical doctor for cardiovascular or neurologic history, addiction, pregnancy and high level sport practicing and were not included (*n* = 1) accordingly. None of the subjects were taking medication. The subjects were instructed to refrain from exercising, drinking alcohol, coffee or tea, and sleep deprivation for at least 24 h before their participation. The study was approved by the Ethical Committee of the University of Aix-Marseille and was conducted in accordance with the Declaration of Helsinki. Written consent was provided by each subject. The subjects performed the study protocol as illustrated in Figure 1.

### Long Duration Switching Task Procedure

The subjects had to perform a task based on the task-switching paradigm during 1 h (Jersild, 1927). We used a custom version of alternating runs based on Rogers and Monsell (1995). Because we aimed to preserve the performance of our subjects on a long run, stimuli were displayed until the subject responded or 2 s. The aim of each trial was to identify accurately and as quickly as possible a letter randomly selected among 16 colored (Blue or Red) letters (AEOUGKMR) displayed on a 2^∗^2 grid on a computer screen (Peirce, 2008) with Psychopy (Open source, University of Nottingham, United Kingdom). The subjects had to identify both the color and nature (vowel or consonant) of the displayed letter. Each trial was constituted as follows: a 2^∗^2 grid pattern appeared immediately at the beginning of the trial, and after 500 ms, a colored letter appeared in one of the four boxes. When the letter appeared on the upper part of the 2^∗^2 grid pattern, the subjects had to push the right arrow keyboard key if the letter was red or the left arrow key if the letter was blue; when the letter appeared in the lower part, the subjects had to push the left arrow key if the letter was a vowel and the right arrow key if the letter was a consonant, regardless of the color. The letters were presented for a maximum 2 s, and the subjects had to press the adequate keyboard key within this time limit. The absence of a response was assimilated to and considered as a wrong response. Following the letter presentation, regardless of whether there was a response, a new trial was initiated after a random interval (200, 450 or 700 ms), while the 2^∗^2 grid pattern remained displayed. The task automatically stopped after 1 h or if the error rate outran 49% starting at 30 min of practice. The first 100 trials were not taken into account; however, they were used as training, and feedback was presented at the end of each trial. The performance feedback disappeared from the 101st trial and then the countdown began.

### Measurements

#### Mental Workload

Objective assessment of mental workload was performed throughout the entire switching task, measuring the mental performance through the calculation of the switching costs for accuracy and response time (Rogers and Monsell, 1995). Four periods (P1, P2, P3, and P4, respectively) of 100 trials (approximately 5 min) were extracted from the 1-h duration task starting at the 10th, 25th, 40th and the 55th min, respectively. These periods were determined to be equally time spaced during the whole protocol duration, to coincide with the end of the temporal quartile of the protocol duration, and to be synchronic with the 5 min cardiovascular recordings necessary for HRV measurements. No performance feedback was provided during these periods.

The visual analog scale (VAS) for fatigue, drowsiness and anxiety was performed before and after the switching task to evaluate the impact experienced by subjects. The NASA-TLX rating scale was completed at the end of the experiment. The NASA TLX rates perceived workload in order to assess a task and consists in 6 subjective subscales rating within a 100-points different aspects of the task. As a multidimensional scale, the NASA-TLX is divided into 6 components with specific subscales as follows: mental demand, physical demand, temporal demand, performance, effort, and frustration (NASA Sc1, NASA Sc2, NASA Sc3, NASA Sc4, NASA Sc5, and NASA Sc6, respectively). As discussed by Hart (2006), we used the raw score of each of the subscales of the NASA-TLX (no pairwise comparisons, no weighting process, no addition), which are more stable and valid metrics.

#### Cardiovascular Function

Room experiment was quiet and its temperature kept constant. Subjects were asked to seat in the comfortable office chair installed in front of the personal computer provided for the experiment. Subjects were asked to relax with free breathing. All experiments started at 10 AM. Three lead electrocardiogram (ECG) and arterial pulse wave were non-invasively (surface thoracic ECG and digital photoplethysmography, respectively) acquired (CNAP Monitor 500, version 3.7.2., CNSystems Medizintechnik AG, Graz, Austria) during the 15 min before the task started (resting control conditions) and during the whole task. The data were then digitized and stored on a personal computer for further analysis. Five periods of 5 min were extracted and analyzed: 5 min before the end of the resting control conditions (Control) and 5 min starting from the 10th, 25th, 40th and 55th min of the task (P1, P2, P3, and P4, respectively). The P1 to P4 periods corresponded to the periods analyzed for the switching costs and performance assessment.

### Cardiovascular Data Analysis

All cardiovascular data analysis was performed from the 5 min beat-to-beat time series constituted from inter-beat intervals from ECG R waves, i.e., the RRI and systolic/mean/diastolic blood pressure (SBP, MBP, and DBP, respectively) that were extracted from raw signals. Raw signals were firstly visually checked and corrected for artifacts (including ectopic beat management, i.e., detection, cancelation, and interpolation) when necessary. Non-evident non-stationarity, such as very slow drifting of the mean or sudden changes of the variance, was observed after linear detrending. No additional filtering techniques such as integration was used. Raw signals were sampled at 1,000 Hz and digitized with a 24-bit analog-to-digital converter. Concerning ECG, Pan and Tompkins real-time QRS detection algorithm was used to automatically detect R-waves and build the RRI series. Discrete original RRI series were resampled by cubic spline interpolation with a 4 Hz sampling rate to generate equidistantly sampled time series *x*(i), *i* = 1, 2,…, *N*.

The HRV was assessed according to the consensual standards (Task Force of the European Society of Cardiology and the North American Society of Pacing and Electrophysiology, 1996). We used Kubios software (Kubios HRV, 2.1, Biosignal Analysis and Medical Imaging Group, Kuopio, Finland).

#### Time-Domain Analysis of HRV

Several classical metrics were used as indexes of the total variability that arises from both random and periodic sources (Billman, 2011), including: the mean RRI and heart rate (HR), the standard deviation of the RRI (STD RRI) and HR (STD HR), the square root of the mean squared differences between adjacent normal RRI (RMSSD), the count of successive normal beat lengths that differed more than 50 ms (NN50), the percentage of successive normal beat lengths that differed more than 50 ms (pNN50), the RRI triangular index, and the triangular interpolation of the discrete distribution of the normal RRI (TINN).

#### Frequency-Domain Analysis of HRV

Different metrics were used to characterize the periodic oscillations of the studied time series using the estimated power spectrum by fast Fourier transform Welch’s periodogram technique. These metrics included the centroid frequency (cf expressed in Hz) and power (pw expressed in ms^2^) in 3 frequency bands of interest: high frequencies (HF, 0.15–0.4 Hz), low frequencies (LF, 0.04–0.15 Hz), and very low frequencies (VLF, 0.003–0.04 Hz). The total power (TP) and LH_pw_/HF_pw_ ratio were also computed. The HF and LF powers were also expressed as the percentage of TP (HF_perc_ and LF_perc_, respectively) and normalized units (HF_nu_ and LF_nu_, respectively) to better reflect the sympatho-vagal components of the HRV and were defined as HF_nu_ = HF_pw_/(TP-VLF_pw_) × 100 and LF_nu_ = LF_pw_/(TP-VLF_pw_) × 100, respectively. These variables were used (Akselrod et al., 1981; Billman, 2011) as indexes of the total variability of the heart rate (TP), the modulation of the sinus node activity by the parasympathetic component of the ANS (HF_nu_), the modulation of the sinus node activity by both the parasympathetic and sympathetic components of the ANS (LF_nu_), the influence of the temperature, renin-angiotensin system and other humoral and hormonal factors on the heart rate (VLF_pw_), and the sympathetic/parasympathetic balance (LF/HF).

#### Informational Domain Analysis of HRV

Most informational domain metrics are known to catch and highlight non-linear properties of the studied time series. We plotted the RRI of rank *n* + 1 as a function of the RRI of rank *n* (lag 1 Poincaré plot) and calculated its SD1, SD2 and SD1/SD2 defined as the standard deviation of the instantaneous beat-to-beat RRI variability (minor axis of the fitted ellipse), the standard deviation of the continuous long-term RRI variability (major axis of the fitted ellipse) and the axis ratio, respectively (Hoshi et al., 2013). They were respectively used as non-linear indexes of (1) the rapid changes in the RRI and thus of the parasympathetic sinus node control (Mourot et al., 2004), (2) the effects of both parasympathetic and sympathetic components on the sinus node activity (De Vito et al., 2002), and (3) the relationship between these components, which is the ratio of the short interval variation to the long interval variation (Acharya et al., 2002). We also used other non-linear tools to characterize the RRI dynamics. Recurrence plot analysis quantification (Mean line length, Max line length, Recurrence rate (REC), Determinism (DET) and Shannon entropy) was performed with embedding dimension, lag, and threshold distance set to *m* = 10, *τ* = 1, and $r=\sqrt{mSD}$ of the RRI time series analyzed, respectively. We computed detrended fluctuation analysis α1 and α2 coefficients with segment length set to *n*∈(4,16) and *n*∈(16,64), respectively. Approximate Entropy (ApEn) and Sample Entropy (SampEn) estimates of each RRI time series were computed with m (embedding dimension) and *r* (filtering level) set to 2 and 0.2 SD of the RRI time series analyzed, respectively.

However, because non-linear deterministic measures of heartbeats have recently been shown to be more sensitive than the stochastic indexes in detecting the autonomic changes related to mortality and because what the time-dependent non-linear metrics show as indicators of cardiac vulnerability to lethal arrhythmias are transient non-stationary shifts of dimension of the heartbeat dynamics to a low value, we ultimately evaluated the correlation dimension (D2) of the RRI times series. D2 quantified the time self-similarity of a signal, i.e., of the RRI time series. D2 can be considered as an estimation of the lower threshold of the number of degrees of freedom for the underlying system generating the observed data and is typically used as an index of the overall complexity of the system dynamics estimated from a time series (Sammer, 1998). D2 was computed as described by Grassberger and Procaccia (1983) with *r* = 15% from the standard deviation of the RRI. Accordingly, the first step is to construct the correlation integral (*N,m,r*) function. The correlation integral counts the fraction of pairs (*X*_i_, *X*_j_) whose distance is smaller than r and is defined as:

$$C(N,m,r)=\frac{1}{N\left(N-1\right)}\overset{N}{\underset{i=1}{\Sigma }}\overset{N}{\underset{j=i+1}{\Sigma }}H\left(r-|xi-xj|\right)$$

*X*_*i*_ and *X*_*j*_ represent phase-space trajectory points, *N* represents the total amount of phase-space points, and *H* represents the Heaviside step function, i.e., *H*(α) = 0 if α < 0 and *H*(α) = 1 if α ≥ 0. D2 is subsequently computed as:

$$D2=\frac{\underset{r\to 0}{\lim}\log\left(C\right)}{\log\left(r\right)}$$

Finally, the baroreflex function assessment consisted of evaluating the set-point (averaged RRI and averaged SBP over 5 min subsequently represented on an *x-y* plot of the SBP-RRI) and the sensitivity (BRS) of the spontaneous arterial baroreflex. The baroreflex sensitivity was calculated as the ratio of the standard deviation of the RRI by the standard deviation of the SBP that has been demonstrated to be a robust estimator of the spontaneous arterial baroreflex sensitivity when compared to 6 other established methods (Bernardi et al., 2010).

To assess the myocardial oxygen consumption, we calculated an indirect index using the double product (SAP × HR), also referred to as the systolic pressure-rate product (Nelson et al., 1974).

### Statistics

Statistics were performed using R statistical software (R Core Team, 2018) with additional packages including lme4 (Bates et al., 2015), car (Fox and Weisberg, 2019), Multcomp (Hothorn et al., 2008). Normality of variables distribution was checked and all the variables used meet the assumptions for the analysis performed. All descriptive data are presented as the mean ± standard deviation. For each cardiovascular variable, five periods were determined: Control, before the task started, and the first, second, third, and fourth temporal quartiles of the task (i.e., from 0 to the 15th min, from the 15th to the 30th min, from the 30th to the 45th min, and from the 45th to the 60th min). For each period, the 5 last minutes of the data were analyzed and will be referred as the Control, P1, P2, P3, and P4, respectively.

To identify the cardiovascular parameters that were altered during mental workload, we performed the following steps. First, linear mixed models and effects were respectively performed and fitted on each measure (Bates et al., 2015), as well as calculated cardiovascular variables (42 cardiovascular variables generated 42 models, i.e., one model per cardiovascular variable) to highlight their modifications during the switching task. The formula for each mixed model was: *Y* ∼ Period + (1| subject), where *Y* is the cardiovascular variable to explain, Period is the categorical fixed effect term (Control and P1 to P4) and subject is the random effect term subject. The form (1| subject) indicates that the model will estimate a random effect intercept for subject. ANOVA tables for each cardiovascular variable were computed on each corresponding linear mixed model using the Wald *F*-test statistics with Kenward-Roger degree of freedom (Fox and Weisberg, 2019) and *post hoc* analysis were performed (Hothorn et al., 2008). Thus, a list of 42 F and corresponding *p*-values was obtained for Period, the fixed effect term. Second, Bonferroni correction was applied on the *p*-value list to control family-wise error rate. From the 42 *p*-values, only cardiovascular models that included cardiovascular variables with significant adjusted *p*-values were selected. Finally, we complementary computed posterior predictive simulation to “look for systematic discrepancies between real and simulated data” (Gelman and Hill, 2006) and used as a diagnostic tool to identify the best of our selected models. The posterior predictive *p*-value is a marker of model robustness (stability across simulations): the higher *p*-value, the higher model robustness.

To quantify statistical links between objective and subjective mental workload quantifiers and cardiovascular HRV parameters, we performed the following steps. First, linear mixed models were used for the task performance variables using the formula: *Y* ∼ Period + (1| subject), where *Y* is each of the task performance variables. Because task was not proposed during the Control period, by definition, the Period variable therefore has only 4 levels (P1, P2, P3, and P4). Linear mixed models were also computed to observe the difference between the VAS scores before and after the task. A final Spearman’s correlation matrix was performed with each regression Period coefficient of the cardiovascular models and the regression Period coefficients of the task performance models, VAS score changes and NASA-TLX subscales scores.

## Results

### Subject Characteristics

Twenty-four subjects were included in the study and performed the task. Nineteen subjects were analyzed (3 subjects were excluded for misunderstanding the task, 1 subject was excluded for lost data, and 1 subject was excluded for highly noisy data). None of the subjects reported mental or physical over activity (sport, work), mental or physical acute or chronic fatigue feelings, or daily medical treatment. Finally, no subjects reported sleep deprivation and drowsiness at the inclusion. The subjects were 25.58 ± 3.24 years old, and their weight, height and BMI were 64.61 ± 12.02 kg, 173.79 ± 8.72 cm, and 21.21 ± 2.27 kg/m^2^, respectively. Nine subjects were female, and nine subjects had regular sports activities (2–4 h per week). Cardiovascular risk factors included a family history of cardiovascular disease or a personal history of cardiovascular disease, including hypertension, angina, and acute coronary syndrome. Seven subjects had at least one cardiovascular risk factor (family history). All patients were also checked for sinus rhythm and a high rate of occurrence of ectopic atrial or ventricular beats.

### Mental Workload

**FIGURE 2:**
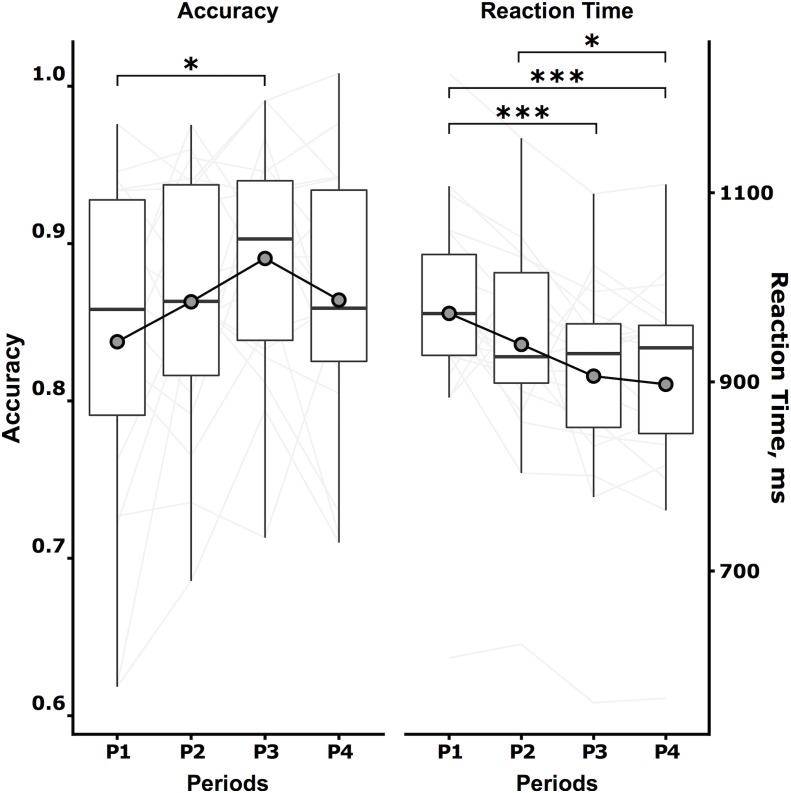
Switching cost kinetics. The switching cost kinetics, i.e., the Accuracy (left, no unit) and Reaction Time (right, milliseconds) during the switching task, are expressed by the boxplot summarizing the data of the 5 last minutes of the four temporal quartiles of the task duration (P1, P2, P3, and P4, respectively). Individuals are represented as light gray continuous lines, and mean data are represented as dark gray circles. Tukey *post hoc* test significance is represented by ^∗^*p* < 0.05 and ^∗∗∗^ *p* < 0.001.

All subjects successfully performed the protocol designed as illustrated in Figure 1 until the end of the task. Switching cost kinetics throughout the protocol are summarized in Figure 2. As indicated, the switching cost on accuracy was impacted throughout the task [*F*(3,54) = 2.98, *p* = 0.039]. The zenith was observed at P3 (P3-P1, estimate = 0.052, *z* = 2.991, *p* = 0.017). No other *post hoc* difference was identified. The switching cost on the response time was also impacted [*F*(3,54) = 9.68, *p* < 0.001]. The nadir was observed at P4 (P4-P1, estimate = –74,996, *z* = 4.818, *p* < 0.001).

The subjective ratings of fatigue and drowsiness were increased by the switching task as shown in Table 1. The switching task led to a relevant mental workload according to the NASA-TLX subscales scores as shown in Table 2. The subjects experienced a true effort characterized by mental, temporal, performance, effort and frustration demands with a similar profile than induced by the “LeeMoray” supervision task (Lee and Moray, 1992).

**Table 1:** Visual Analog Scale for fatigue, drowsiness and anxiety.

Variable	Control	End of task	*p*
fatigue	2.7 ± 2.6	5.0 ± 3.1	<0.000001
drowsiness	2.0 ± 2.4	5.2 ± 3.1	<0.000001
anxiety	2.1 ± 2.5	2.1 ± 2.7	0.939


*The VAS is expressed as the mean (in centimeters) ± SD before (Control) and after (End of) the switching task.*

**Table 2:** NASA-TLX scores.

NASA-TLX subscale	Scores (mean ± *SD*)
NASA Sc1 Mental Demand	55.26 ± 30.07
NASA Sc2 Physical Demand	18.68 ± 14.80
NASA Sc3 Temporal Demand	47.37 ± 25.95
NASA Sc4 Performance	45.26 ± 24.63
NASA Sc5 Effort	56.05 ± 21.25
NASA Sc6 Frustration	43.42 ± 22.92


*The NASA-TLX subscale scores (Sc from 1 to 6) are expressed as the mean ± SD at the end of the switching task according to the assessed dimension.*

### Switching Task Effect on Cardiovascular Variables

#### Selection Step

The global evolution of all cardiovascular parameters during the task and compared to the Control is summarized in Table 3. The switching task led to significant (adjusted *p*-values) changes for 13 cardiovascular variables (family-wise error rate), as shown in Table 4, including descriptors of hemodynamic macro-variables, such as heart rate, blood pressure, and double product, as well as linear and non-linear descriptors of heart rate short-term, long-term, and global dynamics, such as spectral analysis parameters or RMSSD, SD1, SD2, and D2. The subsequent analysis shows that D2 had the highest posterior predictive *p*-value (0.9), nearly double of the mean blood pressure and product (0.465 and 0.457). Interestingly, the posterior predictive *p*-value analysis highlights that all other significantly modified variables by the task had very low posterior predictive *p*-values (<0.3) and extremely low for spectral analysis parameters, such as TP and LFpw.

**Table 3:** Kinetics of cardiovascular parameters during the task.

Variable	Control	P1	P2	P3	P4	*p*-value
**Time domain**						
RRI (ms)	747 (87)	757 (106)	772 (107)	769 (107)	773 (102)	0.018
STD RRI (ms)	57 (27)	39 (14)	43 (14)	49 (189)	51 (21)	0.000037
HR (min^-1^)	82 (9)	81 (11)	79 (11)	80 (10)	79 (10)	0.012
STD HR (min^-1^)	6 (2)	4 (1)	5 (1)	5 (1)	5 (2)	0.000013
RMSSD (ms)	20 (9)	14 (6)	16 (7)	16 (8)	18 (7)	0.00019
NN50 (count)	13 (18)	4 (10)	6 (12)	7 (19)	6 (7)	0.0032
pNN50 (%)	4 (5)	1 (3)	2 (3)	2 (5)	2 (2)	0.0054
RRI triangular index	13 (4)	9 (3)	11 (4)	11 (3)	11 (3)	0.00069
TINN (ms)	276 (127)	188 (56)	216 (75)	226 (75)	260 (97)	0.0001
**Frequency domain**						
VLFcf (Hz)	0.02 (0.01)	0.01 (0.01)	0.01 (0.01)	0.01 (0.01)	0.01 (0.01)	0.25
LFcf (Hz)	0.07 (0.02)	0.08 (0.03)	0.08 (0.03)	0.07 (0.03)	0.08 (0.03)	0.71
HFcf (Hz)	0.17 (0.01)	0.17 (0.02)	0.17 (0.01)	0.17 (0.02)	0.18 (0.03)	0.29
Total power (ms^2^)	2989 (2587)	1876 (1612)	1824 (1527)	2296 (1576)	2740 (2183)	0.029
VLFpw (ms^2^)	1697 (1675)	1203 (1310)	960 (1222)	1415 (1183)	1535 (1534)	0.15
LFpw (ms^2^)	1105 (827)	575 (381)	750 (453)	780 (519)	1051 (848)	0.025
HFpw (ms^2^)	186 (246)	98 (115)	115 (115)	102 (125)	154 (157)	0.025
VLFperc (%)	53 (14)	58 (16)	47 (17)	56 (20)	52 (13)	0.17
LFperc (%)	41 (13)	36 (15)	47 (16)	40 (18)	42 (13)	0.16
HFperc (%)	6 (4)	6 (4)	6 (4)	5 (4)	6 (3)	0.53
LFnu (n.u.)	88 (8)	85 (9)	87 (6)	90 (6)	88 (7)	0.23
HFnu (n.u.)	12 (8)	15 (9)	13 (6)	10 (6)	12 (7)	0.23
LF/HF ratio	10.5 (6.9)	9.2 (6.7)	10.8 (10.5)	13.1 (10.0)	11.4 (13.6)	0.61
**Information domain**						
SD1 (ms)	14 (6)	10 (4)	12 (5)	12 (6)	13 (5)	0.00019
SD2 (ms)	79 (37)	55 (19)	59 (19)	68 (26)	70 (29)	0.000042
Mean line length (beats)	13 (2)	14 (2)	14 (3)	14 (3)	15 (6)	0.11
Max line length (beats)	365 (75)	369 (71)	364 (81)	375 (51)	340 (96)	0.45
REC (%)	39 (5)	41 (5)	40 (6)	41 (5)	43 (10)	0.2
DET (%)	99.51 (0.27)	99.54 (0.25)	99.50 (0.27)	99.58 (0.22)	99.57 (0.24)	0.51
Shannon entropy	3.36 (0.16)	3.41 (0.19)	3.42 (0.21)	3.47 (0.18)	3.48 (0.31)	0.25
alpha 1	1.65 (0.12)	1.59 (0.16)	1.59 (0.17)	1.64 (0.09)	1.64 (0.13)	0.18
alpha 2	1.00 (0.14)	0.99 (0.14)	0.94 (0.18)	0.98 (0.14)	0.97 (0.14)	0.56
ApEn	0.80 (0.09)	0.84 (0.10)	0.82 (0.09)	0.79 (0.10)	0.77 (0.12)	0.033
SampEn	0.83 (0.14)	0.88 (0.16)	0.87 (0.14)	0.80 (0.16)	0.80 (0.21)	0.074
D2	2.21 (0.80)	1.42 (0.85)	1.76 (0.92)	1.82 (0.81)	1.96 (0.90)	0.0002
**Hemodynamics**						
SBP (mmHg)	114 (19)	117 (14)	109 (14)	107 (17)	104 (14)	0.00051
MBP (mmHg)	87 (12)	91 (9)	84 (9)	83 (10)	82 (7)	0.00031
DBP (mmHg)	71 (10)	75 (8)	69 (7)	69 (10)	70 (5)	0.03
DP (bpm⋅mmHg)	8998 (1660)	9197 (1509)	8360 (1254)	8334 (1488)	8096 (1536)	0.00012
Baroreflex sensitivity (bpm⋅mmHg^-1^)	15.3 (7.6)	13.6 (6.6)	13.7 (6.8)	11.9 (7.0)	13.9 (10.9)	0.52


*The kinetics of the cardiovascular parameters during the task are expressed as the mean (SD) throughout the protocol, i.e., during the Control condition and during the last 5 min of the first, second, third, and fourth temporal quartiles (P1, P2, P3, and P4, respectively). RRI, RR Intervals; STD RRI, standard deviation of RRI; HR, heart rate; STD HR, standard deviation of HR; RMSSD, root mean square of successive differences; NN50, number of successive RRI that differ more than 50 ms; pNN50, percentage of successive RRI that differ more than 50 ms; TINN, triangular interpolation of RRI histogram; VLF, very low frequency; LF, low frequency; HF, high frequency; cf, centroid frequency; pw, power; perc, percentage; nu, normalized units; SD1, standard deviation of the instantaneous beat-to-beat inter-beat interval variability (semi-minor axis length of Poincaré plot ellipse fitting); SD2, standard deviation of the long term beat-to-beat inter-beat interval variability (semi-major axis length of Poincaré plot ellipse fitting); REC, recurrence rate; DET, determinism; alpha 1, short-range scaling exponent; alpha 2, long-range scaling exponent; ApEn, approximate entropy; SamEn, sample entropy; D2, correlation dimension; SBP, systolic blood pressure; MBP, mean blood pressure; DBP, diastolic blood pressure; DP, double product. *P*-value of the ANOVA test for Period factor is reported as *p*-value in the last column.*

**Table 4:** Posterior predictive simulation check.

Variable	*p*	adjusted *p*	Posterior predictive *p*-value
STD HR (bpm)	0.0000133	0.000707	0.012
STD RRI (ms)	0.0000366	0.00194	0.00599
SD2 (ms)	0.0000423	0.00224	0.00899
TINN (ms)	0.0001	0.00532	0.0959
Double product (bpm⋅mmHg)	0.000122	0.00649	0.457
RMSSD (ms)	0.000194	0.0103	0.161
SD1 (ms)	0.000194	0.0103	0.174
**D2**	**0.000204**	**0.0108**	**0.9**
MBP (mmHg)	0.000313	0.0166	0.465
LFpw (ms^2^)	0.000385	0.0204	0.015
TP (ms^2^)	0.000396	0.021	0.000999
SBP (mmHg)	0.000514	0.0273	0.236
RRI triangular index	0.000689	0.0365	0.271


*The prediction of the mental workload from cardiovascular variables was assessed by the posterior predictive *p*-value. The highest *p*-value indicates the most predictive variable (bold). All cardiovascular variables were first tested for the period factor fixed effect, and only significant variables are sorted by increasing *p*-values. STD HR, standard deviation of HR; STD RRI, standard deviation of RRI; SD2, semi-major axis of Poincaré plot ellipse fitting; TINN, triangular interpolation of RRI histogram; RMSSD, root mean square of successive differences; SD1, standard deviation of the instantaneous beat-to-beat inter-beat interval variability (semi-minor axis length of Poincaré plot ellipse fitting); D2, correlation dimension; MBP, mean blood pressure; LFpw, low frequency power; TP, total power; SBP, systolic blood pressure.*

#### Effects Throughout the Protocol

**FIGURE 3:**
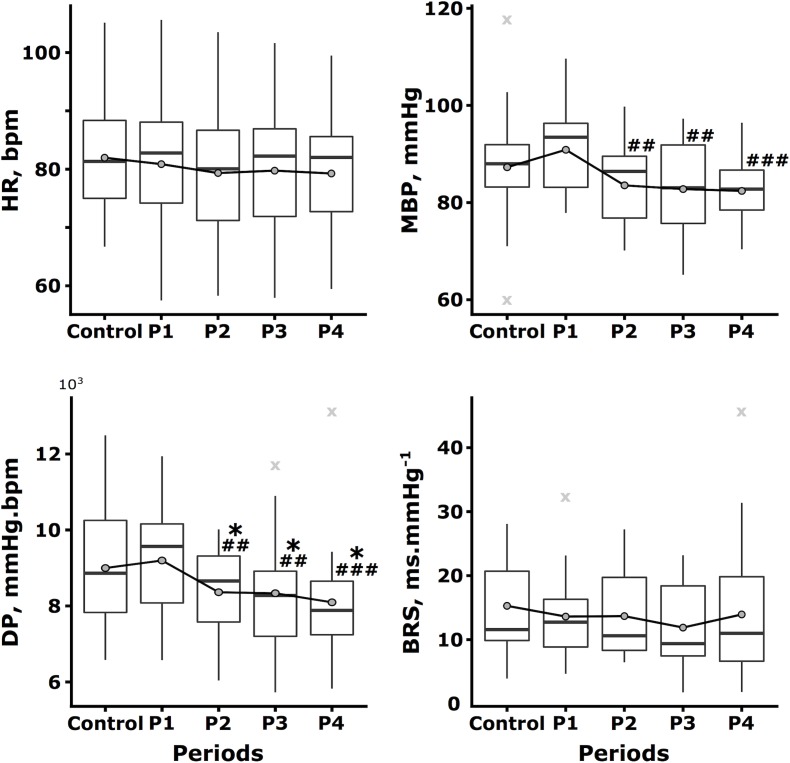
Kinetics of the hemodynamic macro-variables throughout the protocol. The heart rate (HR, bpm), mean blood pressure (MBP, mmHg), double product (DP, mmHg⋅bpm), and baroreflex sensitivity (BRS, ms⋅mmHg^-1^) are expressed by the boxplot represented throughout the protocol, i.e., during the Control condition and during the last 5 min of the first, second, third, and fourth temporal quartiles of the switching task (P1, P2, P3, and P4, respectively). Mean data are represented as dark gray circles and outliers (outlier identification by Tukey’s method) are represented by light gray crosses. Tukey *post hoc* test significance is represented by ^∗^*p* < 0.05 and ^∗∗^*p* < 0.01 compared to the Control, as well as ^##^*p* < 0.01 and ^###^*p* < 0.001 compared to P1.

**FIGURE 4:**
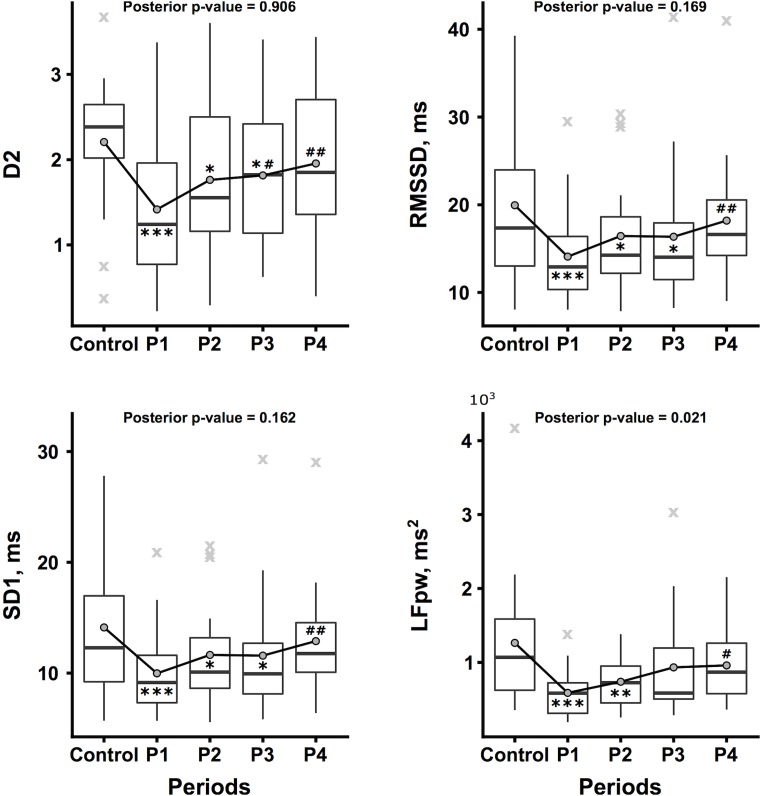
Kinetics of selected heart rate dynamic indexes throughout the protocol. The correlation dimension (D2, no unit), root mean square of successive differences (RMSSD, ms), standard deviation of the instantaneous beat-to-beat inter-beat interval variability (SD1, ms), and low frequency power (LFpw, ms^2^) are expressed by the boxplot represented throughout the protocol, i.e., during the Control condition and during the last 5 min of the first, second, third, and fourth temporal quartiles of the switching task (P1, P2, P3, and P4, respectively). Mean data are represented as dark gray circles and outliers (outlier identification by Tukey’s method) are represented by light gray crosses. Tukey *post hoc* test significance is represented by ^∗^*p* < 0.05, ^∗∗^*p* < 0.01, and ^∗∗∗^*p* < 0.001 compared to the Control, as well as #*p* < 0.05, ##*p* < 0.01, and ###*p* < 0.001 compared to P1.

The kinetics of the hemodynamic macro-variables during the protocol are represented in Figure 3, and the kinetics of selected heart rate dynamic indexes are represented in Figure 4. According to the *post hoc* test results, two different behaviors have been shown as follows: first, the zenith at P1 and a nadir at the Control follow-up by a progressive restoration to the initial level (heart rate family) and second, a zenith was achieved at P1 and a nadir at P4 (blood pressure family).

### Correlation Between Psychometric and Cardiovascular Changes

**FIGURE 5:**
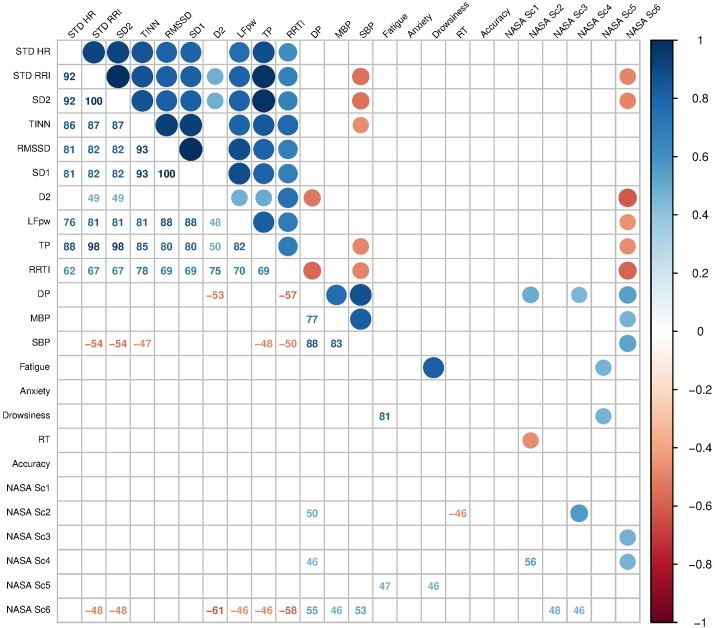
Cardiovascular variable and fatigue index correlations. Matrix correlation of cardiovascular variables and fatigue indexes. Spearman’s coefficient of correlation is expressed by the color scale (up-right) and values (down-left) reported. Selected interesting variables are represented to limit the high dimensional matrix. Fatigue, Anxiety and Drowsiness, visual analog scales of fatigue, anxiety, and drowsiness, respectively; STD HR, standard deviation of HR; STD RRI, standard deviation of RRI; SD2, standard deviation of the long term beat-to-beat inter-beat interval variability (semi-major axis length of Poincaré plot ellipse fitting); RRI TI, RRI triangular index; RMSSD, root mean square of successive differences; SD1, standard deviation of the instantaneous beat-to-beat inter-beat interval variability (semi-minor axis length of Poincaré plot ellipse fitting); D2, RRI correlation dimension; LFpw, low frequency power; TP, total spectral power; DP, double product (HR × SBP); MBP, mean blood pressure; DBP, diastolic blood pressure; SBP, systolic blood pressure; Reaction Time, reaction time to the switching task stimulus; Accuracy, accuracy of the switching task responses. NASA Sc1 to 6, NASA-TLX 1st to 6th subscale scores, respectively. Only significant correlations are shown (*p* < 0.05).

A correlogram plot based on Spearman’s correlation matrix is shown in Figure 5. Only selected cardiovascular variables are shown. The cardiovascular variables were partially correlated with the NASA-TLX scores. In particular, DP was positively correlated with NASA Sc2, NASA Sc4, and NASA Sc6 (*p* = 0.03033, *p* = 0.0494, and *p* = 0.01544, respectively), and D2 was negatively correlated with DP (*p* = 0.02224) and NASA Sc6 (*p* = 0.005459).

## Discussion

### Major Findings

We aimed to characterize the cardiovascular consequences of a calibrated mental workload and focused on the HRV responses. We also searched for a link between the magnitude of the mental workload and cardiovascular changes. A global decrease of the HRV was observed as assessed in time- as in frequency-domains, without any evident dominance of the para- or ortho-sympathetic components of the ANS. The sympatho-vagal balance that regulates HR was unchanged. The HRV decrease was maximum at the beginning of the task and then faded and even disappeared. D2, a non-linear index that does not reflect the neuromodulation of the sinus node activity by the sympatho-vagal balance but time self-similarity of the RRI time series, was decreased during the whole dual task in contrast to the other non-linear HRV parameters. D2 was the most relevant, significant, and robust HRV parameter modified by mental task and workload. D2 was negatively correlated with the NASA Sc6 component (frustration) and double product (myocardial oxygen consumption).

### Mental Workload and HRV

#### Time and Frequency Domain HRV Indexes

Time domain and frequency domain HRV analyses have been widely used to investigate the cardiovascular consequences of mental work, as previously reported by Kramer (1990). Typically, mental workload leads to a decrease of the time domain measures, as well as a decrease of the LH and HF powers, while the LF/HF ratio increases. This supposes a predominant decrease of the parasympathetic activity or a predominant increase of the sympathetic activity (Hjortskov et al., 2004; Wang et al., 2005; Moses et al., 2007; Weippert et al., 2009; Trutschel et al., 2012). The cardiovascular response to mental workload we observed does not evidence a clear sympathetic activation while frequency domain analysis has been reported to be particularly recommended in these situations (Jorna, 1992). Two hypotheses can explain this finding: whether the subjects had a LF/HF ratio that was high prior to the beginning of the task, probably attesting an anxious anticipation phenomenon, or the task, which was repetitive, not performed in real-life but in a laboratory environment, and without a particular issue, could have limited the task-induced anxiety. Our baseline data support the first hypothesis, although the second hypothesis cannot be formally excluded.

#### Non-linear HRV Indexes

To our knowledge, non-linear HRV analysis methods have rarely been used in mental workload conditions. At most, a correlation between SD2 and cognitive performance indexes, i.e., vehicle lane-deviation in a night driving task and variation of the tracking error for a compensatory tracking task, has been found with *r*^2^ = 0.55 and *r*^2^ = 0.73, respectively (Trutschel et al., 2012). It is a positive correlation, which indicates that SD2 increases when performance decreases. We observed a decrease of SD1 and SD2 at the beginning of the task; however, these measures subsequently increased to reach their Control values at the end of the task. SD2 is typically considered as a non-linear index of both parasympathetic and sympathetic neuro-regulation of heart sinus activity.

Concerning D2, few studies are available. Both acute and chronic psychic stress have been shown to lower D2 (Schubert et al., 2009) while previously Sammer (1998) reported that D2 allowed to discriminate between a physical and mental task and that the Lyapunov exponent was related to the amount of the task. The values of D2 were lower in the physical task (approximately 3.7) but two main differences must be highlighted compared to our study. First, Sammer’s study used a short-term task (105s) that may induce a lower mental fatigue than in our task and second, the time series used were clearly too short (105s) to perform HRV spectral analysis according to standards, as well as to perform non-linear analysis. The non-linear analysis of a dynamic system is often limited with short and noisy time series (see “Limits” section).

In our study, D2 was the most impacted cardiovascular parameter in terms of significance as well as kinetics. Concerning significance, the exact physiology that underlies D2 is not established; however, it is supported by the complex systems approach. Time- and frequency- domain measures are considered to reflect the ANS influence on the cardiovascular system, unlike D2. D2 expresses the rate of visitation of areas in the multidimensional space spanned by the data. In our study, the lowest mean value of D2 was at 1.42 ± 0.85 and the mean value of D2 at the end of the task was 1.96 ± 0.9. These values are lower than experiment control D2 values (2.21 ± 0.8), but also than our internal reference values from a similar but distinct 25 healthy subjects group (3.21 ± 1.14) and finally than literature data. In the Skinner’s study (Skinner et al., 1993), only 3 of 25 normal subjects had values below 2.0 and the mean value of the ventricular tachycardia group was 1.3 ± 1.7. Thus, our values could be considered pathologic or at least non-physiological. This result is particularly important as it is established that a decreased D2 can be predictive of the onset of severe arrhythmias in the population of patients at high risk of sudden death. D2 has been shown to be precocious and independent from the other cardiovascular parameter arrhythmic risk factor markers, including time-domain and spectral analysis indexes of the HRV (Skinner et al., 1993). As typical cardiovascular risk factors are not always found to be heralding of sudden cardiac death (Myerburg and Spooner, 2001), the clinical interest of D2 as a complex heart rate dynamic index might be confirmed in a healthy population. In our study, in which the subjects did not exhibit cardiovascular risk factors except tobacco smoking for some subjects, we showed that the heart rate dynamic complexity (cardiac adaptability) was altered according to the mental workload. We could propose the possibility of considering D2 as a monitoring parameter for the cardiovascular impact on the mental workload. Concerning kinetics, we note that D2, as an index of the overall complexity of RRI time series that reflect HR adaptability, reached its minimum value during P1, i.e., at the beginning of the task. This initial drop of D2 could be linked with the learning solicitation that is maximum at the beginning of the task. This hypothesis is supported by our others results. Mental performance as assessed by objective metrics seems to improve throughout the task supporting a clear learning effect: Accuracy increases while Reaction Time decreases. Simultaneously, double product, that is an indirect index of myocardial oxygen consumption, i.e., of cardiac metabolic stress, increases at the very beginning of the task (P1) while it significantly lowers at P2, P3 and P4. This initial burst in cardiac solicitation (significant with paired *t*-test) could be explained as a side-effect of mental strain since DP is correlated to NASA Sc2, Sc4, and Sc6 and fatigue is clear at the end of the task. On the contrary, the delayed decrease in DP is synchronous of the Accuracy increase as the Reaction improvement that could be explained as a benefic effect of learning, the experimental condition being less wounding as drowsiness is highly expressed at the end of the task also. Tasks that do not continuously involve working memory can induce a high mental workload also; however, subjects will be able to reduce their effort during the task without causing an unacceptable drop of performance (Mulder, 1992). In our study, working memory is not involved, except probably at the very beginning of the task to memorize the rules. But the effect of such learning on cardiovascular parameters has been reported by others also (Taelman et al., 2011). The authors studied the heart rate and HRV under a physical task, the same task was then added with a mental task, during 6 min. Spectral analysis of the HRV indicated a decrease in the variability expressed by the time domain parameters and a decrease in the power of all spectral components with the addition of the mental task. This effect was transient and lasted only 3 min. Moreover, the alterations observed in a first session of the double task are less important than the alterations observed in the next session of this task, which further confirms the hypothesis of adaptation. This phenomenon was studied by McEwen and Stellar through the concept of homeostasis: they explain the mechanisms of adaptability in humans to unknown stressful situations (McEwen and Stellar, 1993). Finally, we showed that D2 is negatively correlated to DP, the highest DP, the lowest D2. We can then hypothesize that D2 is a sensitive marker of cardiac metabolic strain modifications induced by the mental workload unlike usual HRV indexes that should be tested in further studies. The usual parameters describing the HRV (from time- and frequency- domain analyses) and D2 are not always linked and correlated. Linear approaches loose a part of their sensitivity when a non-stationary phenomenon occurs, and as the breathing pattern is known to be altered and variable during mental workload (Veltman and Gaillard, 1996; Taelman et al., 2011), we can consider that the neuro-regulation of the HR was not stationary. In our protocol, subjects maintained a free-breathing pattern during the whole task, with the respiratory sinus arrhythmia being possibly not regular nor stationary.

### Limits

#### Sample Size and Population Characteristics

Even if sample size calculation (*n* = 16) led to the theoretical adequate number of subjects to be included (*N* = 24) to have a statistical power of 0.8, only 19 subjects’ data were used. Twelve to twenty subjects are usually relevant in HRV physiology studies, especially when powerful stimuli are used (orthostatism, hypoxia, hypercapnia, or exercise) to disrupt the HR regulation system. However, because of HRV inter-individual as intra-individual high variability and because of the weaker stimulus used here that is mental workload, all results need to be interpreted with caution. Moreover, our work concerned healthy subjects, aged from 20 to 35 years old. They are supposed to have a normal cardiovascular function with a normal HRV. This recruiting bias makes an extrapolation of the results to the general population difficult and even more to the cardiovascular-risked population. Further studies seem necessary to replicate and strengthen our results.

#### Length of the Analyze Time Series

On one hand, the length of RRI time series used to perform time- and frequency- domains HRV analysis is usually set to 5 min (Task Force of the European Society of Cardiology and the North American Society of Pacing and Electrophysiology, 1996). The value of the scaling exponent is mostly defined by the dynamics of the short-time variability. Therefore, it can be used as a certain measure of short-time variability of the signal but records length has to be comparable. On the other hand, and oppositely, exploring the dimensionality of the space spanned by the data, particularly when using D2, requires long time series to be reliably computed (Säkki et al., 2004). Time series are supposed to include 10^D2^ data points, i.e., around 10.000 data points should be used. Using only and approximately 400 data points per time series, we can’t consider that D2 we computed represent robustly and reliably the whole concept and the underlying properties defined by the correlation dimension. But decidedly, the computation we performed (that of D2, i.e., the correlation sum) on 400 data points time series led to a metrics that was statistically characterized as the most sensitive, reliable, and stable marker. Accordingly, we then highlight that the interpretation of the time behavior of the RRI time series and HR dynamics should be made with caution: The D2 changes we measured during the task are not necessarily the results of the changes in the scaling behavior of the heart rate dynamics. Specific studies are now needed to test this hypothesis.

#### Baroreflex Assessment

We found no change in the spontaneous arterial baroreflex sensitivity. We used a simple methodological approach, the ratio between the standard deviations of RRI and SBP, that intercepted the median of standard BRS methods equally or better than any other method, indicating that this index can be used as a reliable method for measuring BRS (Bernardi et al., 2010). Nevertheless, this approach could be not appropriate to really disentangle true baroreflex effects from non-baroreflex or respiration-mediated influences. Further research is needed with appropriate and specific tools targeting specifically baroreflex assessment through causality concept (Porta et al., 2000; Nollo et al., 2005; Faes et al., 2013) before definitively excluding effects on arterial baroreflex.

#### Habituation

Our task’s characteristics and particularly its monotony, as well as the instructions’ constancy might have enabled a habituation phenomenon. Thus, mental workload mainly lies in the attention required during a long time and thereby the struggle against fatigue and drowsiness, substantially more than the cognitive load, which is preponderant in the beginning of the task only. The physical task elicited in our study may have been lower than in other tasks closer to the real practice, and it may contribute to a non-activation of the sympathetic system, as attested by the unchanged sympatho-vagal balance and the non-elevation of heart rate and blood pressure. The design of the ideal study would be a compromise between the duration of the task and the habituation aroused, i.e., a more complex task, for example, that involves the working memory, and/or a longer task duration.

## Conclusion, Contributions and Prospects

We investigated the changes of HRV non-linear indexes during a switching task to search for markers of the cardiovascular consequences of mental work and workload. We showed that the RRI correlation dimension (D2), a non-linear marker of the degree of freedom of the cardiovascular system, was the most sensible and impacted metric by the mental task and workload and was negatively correlated with frustration and cardiac oxygen consumption. Nevertheless, additional research is required to confirm these initial results on the usefulness of D2 as a marker of the impact of mental workload on cardiovascular function.

## Ethics Statement

This study was carried out in accordance with the recommendations of the Ethical Committee of the University of Aix-Marseille with written informed consent from all subjects. All subjects gave written informed consent in accordance with the Declaration of Helsinki. The protocol was approved by the Ethical Committee of the University of Aix-Marseille.

## Author Contributions

SD conceptualized and designed the study, performed the HRV analysis and exploratory data processing, analyzed the results, wrote the manuscript, and led the research. GC designed the study, developed and wrote the switching task procedure, performed extensive data analysis, analyzed the results and wrote the manuscript. AD managed the clinical data acquirement, conducted elementary statistics, and wrote the manuscript. J-CD intensively discussed the results and highly commented the revised manuscript.

## Conflict of Interest Statement

GC was employed by the company AltraBio. The remaining authors declare that the research was conducted in the absence of any commercial or financial relationships that could be construed as a potential conflict of interest.
